# Effect of tDCS stimulation of motor cortex and cerebellum on EEG classification of motor imagery and sensorimotor band power

**DOI:** 10.1186/s12984-017-0242-1

**Published:** 2017-04-19

**Authors:** Irma N. Angulo-Sherman, Marisol Rodríguez-Ugarte, Nadia Sciacca, Eduardo Iáñez, José M. Azorín

**Affiliations:** 1 Center for Research and Advanced Studies (Cinvestav), Parque de Investigación e Innovación Tecnológica km 9.5 de la Autopista Nueva al Aeropuerto, 201, Monterrey, 66600 NL Mexico; 2 Brain-Machine Interface Systems Lab, Universidad Miguel Hernández de Elche, Av. de la Universidad S/N, Elche, 03202 Spain

**Keywords:** tDCS, Rehabilitation, EEG, Motor imagery

## Abstract

**Background:**

Transcranial direct current stimulation (tDCS) is a technique for brain modulation that has potential to be used in motor neurorehabilitation. Considering that the cerebellum and motor cortex exert influence on the motor network, their stimulation could enhance motor functions, such as motor imagery, and be utilized for brain-computer interfaces (BCIs) during motor neurorehabilitation.

**Methods:**

A new tDCS montage that influences cerebellum and either right-hand or feet motor area is proposed and validated with a simulation of electric field. The effect of current density (0, 0.02, 0.04 or 0.06 mA/cm^2^) on electroencephalographic (EEG) classification into rest or right-hand/feet motor imagery was evaluated on 5 healthy volunteers for different stimulation modalities: 1) 10-minutes anodal tDCS before EEG acquisition over right-hand or 2) feet motor cortical area, and 3) 4-seconds anodal tDCS during EEG acquisition either on right-hand or feet cortical areas before each time right-hand or feet motor imagery is performed. For each subject and tDCS modality, analysis of variance and Tukey-Kramer multiple comparisons tests (*p* <0.001) are used to detect significant differences between classification accuracies that are obtained with different current densities. For tDCS modalities that improved accuracy, t-tests (*p* <0.05) are used to compare *μ* and *β* band power when a specific current density is provided against the case of supplying no stimulation.

**Results:**

The proposed montage improved the classification of right-hand motor imagery for 4 out of 5 subjects when the highest current was applied for 10 minutes over the right-hand motor area. Although EEG band power changes could not be related directly to classification improvement, tDCS appears to affect variably different motor areas on *μ* and/or *β* band.

**Conclusions:**

The proposed montage seems capable of enhancing right-hand motor imagery detection when the right-hand motor area is stimulated. Future research should be focused on applying higher currents over the feet motor cortex, which is deeper in the brain compared to the hand motor cortex, since it may allow observation of effects due to tDCS. Also, strategies for improving analysis of EEG respect to accuracy changes should be implemented.

## Background

Transcranial direct current stimulation (tDCS) is a noninvasive technique for brain stimulation where direct current is supplied through two or more electrodes in order to modulate temporally brain excitability [1, 2]. This technique has shown potential to improve motor performance and motor learning [3–5]. Hence, it could be applied in motor neurorehabilitacion [1]. However, tDCS effects vary depending on several factors, such as the size or position of the stimulation electrodes and the current intensity that is applied [6] or the mental state of the user [7]. Therefore, it should be considered that outcomes of tDCS studies are the result of different affected brain networks that may be involved in attention and movements, among other processes.

Volitional locomotion requires automatic control of movement while the cerebral cortex provides commands that are transmitted by neural projections toward the brainstem and the spinal cord. This control involves predictive motor operations that link activity from the cerebral cortex, cerebellum, basal ganglia and brainstem in order to modify actions at the spinal cord level [8]. In general, this set of structures can be considered to form a motor network that allow voluntary movement.

Different parts of the cerebral cortex participate in the performance of self-initiated movement, like the supplementary motor (SMA), the primary motor (M1) and premotor (PM) areas. It is known that M1 is activated during motor execution. Excitatory effects of M1 have been studied with anodal stimulation [6], finding that activation of this region is related to higher motor evoked potentials (MEPs) and an increment of force movement on its associated body part area [9, 10]. Moreover, M1 seems to be critical in the early phase of consolidation of motor skills during procedural motor learning [11], i.e., the implicit skill acquisition through the repeated practice of a task [12].

In addition, the SMA and PM influence M1 in order to program opportune precise motor commands when movement pattern is modified intentionally, based on information from temporoparietal cortices regarding to the body’s state [8]. The SMA contributes in the generation of anticipatory postural adjustments [13]. Consequently, its facilitatory stimulation seems to increase anticipatory postural adjustments amplitudes, to reduce the time required to perform movements during the learning task of sequential movements, and to produce early initiation of motor responses [14–16]. These studies suggest the possibility of using SMA excitation during treatments for motor disorders, since hemiparesis after stroke involves the impairment of anticipatory motor control at the affected limb [17]. In addition, some studies propose the participation of the SMA in motor memory and both implicit and explicit motor learning [18–21], i.e, when new information is acquired without intending to do so and when acquisition of skill is conscious [22], respectively. Complimentary to the role of SMA, the PM is crucial for sensory-guided movement initiation and the consolidation of motor sequence learning during sleep [8, 23], while its facilitation with anodal tDCS seems to enhance the excitability from the ipsilateral M1 [24], which may be useful for treatment of PM disorders.

As previously mentioned, the cerebellum is also involved in locomotion through the regulation of motor processes by influencing the cerebral cortex, among other neural structures. During adaptive control of movement, as in the gait process, it seems that loops that interconnect reciprocally motor cortical areas to the basal ganglia and cerebellum allow predictive control of locomotion and they exhibit correlation with movement parameters [8, 25]. Regarding to studies about cerebellar stimulation, there is still not enough knowledge about the effects of tDCS on different neuronal populations and the afferent pathways, so results are variable among studies and their interpretation is more complex than for cerebral tDCS [26]. Furthermore, the topographical motor organization of the cerebellum is not clear yet [27]. Nevertheless, most studies base their experimental procedure on the existence of decussating cerebello-cerebral connections, even if there are also ipsilateral cerebello-cerebral tracts or inter-hemispheric cerebellar connections [28]. Hence, a cerebellar hemisphere is stimulated to affect cerebellar brain inhibition (CBI), which refers to the inherent suppression of cerebellum over the contralateral M1 [29]. For example, the supply of anodal and cathodal stimulation over the right cerebellum in [30] resulted in incremental and decremental CBI on the left M1, respectively. In contrast, there are some studies that suggest this expectation may be not always appropriate. In [31] it was shown that inhibitory transcranial magnetic stimulation (a stimulation technique that provides magnetic field pulses on the brain [32]) over the lateral right cerebellum led to procedural learning decrement for tasks performed with either the right or left hand, whereas inhibition of lateral left cerebellar hemisphere decreased learning only with the left hand. In addition, results from [33] showed that cathodal cerebellar tDCS worsened locomotor adaptation ipsilaterally. These two studies may provide a reference for using cerebellar inhibition for avoiding undesired brain activity changes during motor rehabilitation, such as compensatory movement habits that might contribute to maladaptative plasticity and hamper the goal of achieving a normal movement pattern [34].

Even though, as mentioned in [35], deep brain structures cannot be targeted directly with noninvasive stimulation strategies like tDCS, some parts of the neural circuitry could be targeted with this kind of stimulation. For this purpose, diffusion MRI tractography, which is a noninvasive method for visualizing brain connectivity, could be useful. Despite its limitation on anatomical accuracy, diffusion MRI tractography may offer a guide for targeting white matter pathways [36]. In addition, this method has been already used to describe the cerebello-cerebral pathway, showing connections at the cerebellum, red nucleus, thalamus, and motor cortex [37]. Since tDCS results probably depend on the direction of the current flow respect to neuronal orientation [38–40], it can be hypothesized that orienting the electric field similarly as followed in the cerebello-cerebral tract may improve reproducibility of results.

Mental practice of specific motor tasks elicits the activity of part of the network within the motor control system [2]. For this reason, during both movement performance and its mental practice, there is attenuation of *μ* (8-12 Hz) and *β* (13-30 Hz) band power on electroencephalographic signals (EEG) [41] due to the desynchronization of neuronal activity at these frequencies. Hence, motor imagery (MI) is included in some experimental protocols focused on motor neurorehabilitation [42, 43], such as studies that use brain-computer interfaces for future therapies [44]. Note that the participation of subcortical structures is also observed in functional imaging studies during mental imagery [45].

Based on all these studies, it can be inferred that the stimulation of different parts of motor network may be useful in motor neurorehabilitation since brain conditions of stroke patients are heterogeneous in terms of the site and size of the possible lesions [46]. Hence, in this study it is proposed a montage approach that is aimed to stimulate different regions involved in locomotion: M1, PM, SMA and cerebellum, with the objective of targeting parts of the cerebello-cerebral pathway and establishing the current flow with a relatively similar orientation than the one at some portions of the tract [37]. In particular, this study proposes and evaluates a new tDCS montage that affects both the cerebellum and the feet or right-hand motor area to test its suitability for MI-based BCIs and motor neurorehabilitation. For this purpose, the proposed montage was first validated with simulations of the electric field that is produced by the montage. Then, the effect of current density on EEG classification into rest state or either right-hand or feet MI was assessed for three stimulation modalities: applying 10-minutes anodal tDCS before EEG acquisition over the cortical area that is associated with 1) right hand or 2) feet movement, and 3) stimulating with 4-seconds anodal tDCS during EEG recording either on right-hand or feet cortical areas before each performed right-hand or feet MI, respectively. This would allow determining the current range that may be needed to enhance MI detection with the proposed montage. Also, *μ* and *β* band power for tDCS modalities that could improve classification was compared with the no stimulation sessions in order to obtain an insight about the possible EEG changes that may lead to classification improvements.

The study here presented is part of the Associate Project - Decoding and stimulation of motor and sensory brain activity to support long term potentiation through Hebbian and paired associative stimulation during rehabilitation of gait (DPI2014-58431-C4-2-R), funded by the Spanish Ministry of Economy and Competitiveness and by the European Union through the European Regional Development Fund “A way to build Europe”, in which different approaches are being conducted in order to evaluate the potential positive results derived from a new tDCS montage upon motor rehabilitation. The proposed montage disposition is aimed to improve motor performance of both lower and upper limbs by enhancing the excitability of stroke patients’ motor pathways.

## Methods

### Experimental set-up

#### EEG acquisition equipment

The Enobio system (Neuroelectrics) is used to acquire EEG from 32 channels (P7, P4, CZ, PZ, P3, P8, O1, O2, C2, F8, C4, F4, FP2, FZ, C3, F3, FP1, C1, F7, OZ, PO4, FC6, FC2, AF4, CP6, CP2, CP1, CP5, FC1, FC5, AF3, and PO3) according to the international 10/10 system at a sampling rate of 500 Hz.

#### tDCS supply

**Fig. 1:**
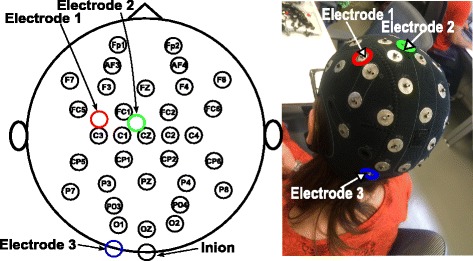
tDCS montage. Scheme of tDCS electrodes position in reference to EEG electrodes and inion (*left*), and placement of tDCS electrodes on the EEG cap (*right*). Electrodes 1,2 and 3 are highlighted in *red*, *green* and *blue*, respectively

Two or three stimulation channels of the Starstim system (Neuroelectrics) are used to supply anodal tDCS through electrodes with 1 cm radius, depending on the evaluated tDCS modality. These electrodes are denoted as electrodes 1, 2 and 3. Electrodes 1 and 2 represent the anode position for two possible versions of the montage approach that are used to provide different stimulation modalities in which either the right-hand or feet motor area is stimulated, while electrode 3 is the cathode in both montage versions. Electrode 2 is placed on the midpoint of Cz and FC1 in order to stimulate the SMA and feet motor cortex. In contrast, electrode 1 is located in front of C3, after an imaginary line that is traced from C1 to FC5. This electrode is aimed to stimulate right hand motor and premotor areas, while keeping the distance to C3 similar to the one found between electrode 2 and Cz. Electrode 3 is positioned at the inion level, but displaced approximately 3 cm to the left hemisphere. This location was selected based on [47], which states that various studies of cerebellar tDCS place the stimulating electrode 3 cm lateral to the inion, which is close to cerebellum. These stimulation electrodes are shown in Fig. 1.

This way, the proposed montage version that targets feet motor area, attempts to increase the excitability of the SMA and feet M1, which is expected to increase feet motor performance and improve the initiation of motor activity, regardless of the kind of movement due to the broad stimulation of SMA. In contrast, the montage version that targets right-hand motor area is expected to improve right-hand motor performance by exciting right-hand M1 and PM. Both montage versions would inhibit left cerebellum, considering that the cathode is placed over it. Since the position of electrode 3 (displaced 3 cm lateral to the inion) is also used in [33] for decreasing ipsilateral motor adaptation, we hypothesized that task-adaptation would be decreased for the left limbs. However, it could be expected also the possible facilitation of the right motor cortex (associated to the left limb) due to CBI suppression.

#### tDCS simulation

SimNIBS free platform [48] is employed in order to produce two simulations of the electric field that is produced by the proposed tDCS montage on a standardized healthy brain: one including electrodes 1 and 3, and another with electrodes 2 and 3. Current values represented approximately the maximum current that is used on the experiments, i.e. 188 *μ*A. In particular, the current in the simulations is configured as 180 *μ*A. Electrodes are modeled with radius of 1 cm, thickness of 3 mm and a space for conductive gel of 4 mm, based on measures of the electrodes that are used in the experiments.

#### Subjects

Five volunteers (two male and three female between 27 and 35 years old) without metallic implants or known neurological disorders participated in this study. Subjects were labeled as S1, S2, …, S5.

#### Experimental procedure

Each subject went through twelve experimental sessions performed in different days, leaving between sessions at least two days to allow recovery from tDCS effects. This recovery interval was selected taking into account that an intersession interval of 48 hours to one week is recommended for protocols with long-lasting effects of more than one hour [6]. Since aftereffects duration has not been evaluated yet for the proposed montage, it was assumed that the duration would be similar to the one obtained with another cephalic montage for stimulating the motor cortex [49]. With such montage, the effects of stimulating with a current intensity of 1 mA and an electrode size of 35 cm^2^ (an approximate current density of 0.03mA/cm^2^) for nine and eleven minutes provided significant aftereffects for up to thirty and fifty minutes, respectively.

**Fig. 2:**
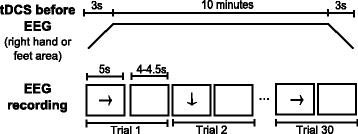
Temporal sequence when tDCS is provided before EEG recording. Series of events in time for stimulation (*top*) and a run (*bottom*) when tDCS is provided before EEG recording
- **Supply of tDCS on the brain region related to feet movement before EEG recording**:
  The procedure for this modality is similar to the previous one, with the only difference of using electrode 2 as anode and that the feet motor region of the brain is stimulated instead of the hand area.
- **Supply of tDCS on the brain region related to right-hand or feet movement during EEG recording**:
  In this tDCS scheme, the subject looks at a screen that can show three possible visual cues: an arrow, a blank screen (both with the same meaning as in the two previous modalities) or the word “Stimulation”. When the latter appears, tDCS is applied over the brain motor area that is associated with the next visual cue, which is one of MI. This means that before displaying an arrow directed toward the right or the bottom, tDCS is administered using electrode 3 as cathode and electrode 1 or 2 as anode, respectively. The visual cue of stimulation lasts 16 s, although the tDCS pulse actually has a duration of 4 s with a ramp of 3 s at the beginning and the end of stimulation, as can be observed in Fig. 3. This is done because the activation and deactivation of the Starstim device have a delay of variable duration that introduces noise in the EEG, so these time lapses have to be discarded. Moreover, the rest cue lasts 4 s more in comparison to other modalities as an effort to dissipate the tDCS effects during the first seconds of the cue. This extra time was selected considering that tDCS of 4 s and current density of 0.029 mA/cm^2^ with another cephalic montage [3] needs just a break of 10 s to avoid tDCS effects. Even though the duration of the effect depends on the montage, this was the only reference for the unknown value of the persistence of the effect.

The twelve sessions were done in three blocks of four sessions where it was evaluated only one of the following stimulation modalities:

- **Supply of tDCS on the brain region related to right-hand movement before EEG recording**:
  First, anodal tDCS is applied for 10 min with 3-seconds ramps at the beginning and the end of stimulation. Electrode 1 works as anode, while electrode 3 as cathode. Then, EEG is recorded while the subject stares at a screen that shows sequences of instructions. If the screen is cleared (4 to 4.5 s), the user has to remain at rest. On the other hand, if an arrow pointing to the right or down appears (5 s), the subject has to imagine to move the right hand or feet, respectively. Every session consists of three runs of fifteen sequences or trials of each kind of motor imagery (right hand or feet) plus a corresponding period of rest, i.e., each run includes the random presentation of fifteen trials of right hand motor imagery and fifteen trials of feet motor imagery. Figure 2 shows the temporal sequence of tDCS and a run for this modality, in which a total of thirty trials (motor imagery plus rest) are recorded. Between runs there are given breaks of 3 min.

**Fig. 3:**
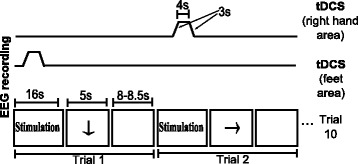
Temporal sequence when tDCS is provided during EEG recording. Series of events in time for stimulation (*top*) and a run (*bottom*) when tDCS is provided simultaneously to EEG recording
  In this modality, every session consisted of nine runs of five trials of both kinds of MI, with their associated rest time. There were breaks of one minute between the runs. Note that the short stimulation duration from this tDCS modality is not considered to induce long-lasting aftereffects. Then, it is unlikely that it produces neuroplasticity due to cumulative effects, but maybe only for strong current intensities that extend their effects to overlap with the next stimulation pulse. The main interest in implementing this stimulation modality is to evaluate the changes in classification when the tDCS is applied only to increase excitability during MI and the EEG band power changes are compared to the rest state without any excitability effect, which would allow short-time comparisons in band power. This approach could have application in just enhancing MI detection during training so EEG changes are better detected. However, this is not expected to reduce the required training sessions to induce plasticity in neurorehabilitation, which would make the use of tDCS inefficient. On the other hand, MI detection enhancement could be useful for improving the detection of individualized EEG features for proper calibration of systems for brain entrainment before training is started.

**Fig. 4:**
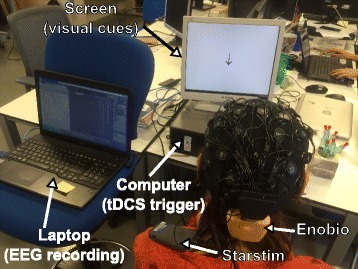
Experimental setup. The experimental setup consists in the user wearing an EEG cap, the Enobio system and the Starstim device, while watching visual cues on a screen. A laptop that records EEG is able to control cues and activate Starstim in other computer

In every block of tDCS modality, each of the four sessions had a different current density value that was assigned in random order: 0 (or sham), 0.02, 0.04 or 0.06 mA/cm^2^. Also, the order of evaluation of tDCS modalities was counterbalanced between users. Sham stimulation was used only when tDCS was applied before EEG adquisition. In contrast, when tDCS pulse lasted 4 s the current intensity analog to sham was just configured as 0 mA/cm^2^ for practical reasons. In addition, the lowest current density value was set as 0.02 mA/cm^2^ in order to have a reference that was close to 0.028 mA/cm^2^, which is the recommended maximum limit of current density [50]. Despite this recommendation, most tDCS studies evaluate current densities between 0.029 and 0.08 mA/cm^2^ [6]. Hence, maximum current density was defined as 0.06 mA/cm^2^. Figure 4 shows the experimental setup that was used in this study.

Considering that current density is estimated as the ratio of current intensity and the electrode area [6], the current values required to produce the current densities of 0.02, 0.04 or 0.06 mA/cm^2^ are 63, 126 and 188 *μ*A, respectively. This estimation of the current density can be inaccurate for small electrodes [51]. However, [52] reported that the reduction of ten times of the electrode size produced no significant difference in the elicited effect of tDCS if the current density was kept constant in a cephalic montage. In particular, the effects obtained with electrodes of 35 cm^2^ and 1000 *μ*A were similar to the case when 100 *μ*A were used with electrodes of 3.5 cm^2^, which is an electrode area close to the one of the present study (*π* cm^2^). Therefore, the ratio of current intensity and electrode size was used in the study to calculate the current density.

#### EEG processing and analysis

On each session, the percentage of correct classifications (i.e., accuracy) for each kind of MI and rest is obtained. The changes on event-related syncronization (ERS) for *μ* and *β* bands, which is the increment on power related to a task, is also calculated for those tDCS modalities that show an improvement in accuracy when stimulation is applied. All the EEG processing required to obtain accuracy and ERS, as well as their further analysis was performed using MATLAB software [53].

In order to obtain the accuracy and power in one session, the EEG signals are first preprocessed with the aim of getting EEG signals that are clean from artifacts for further analysis. The initial part of preprocessing consists in filtering EEG signals with a fourth-order bandpass Butterworth with cut-off frequencies of 5 and 45 Hz. Then, each trial undergoes independent component analysis (ICA) with EEGLAB toolbox [54] in order to detect visually the presence of blinking artifacts. When an artifact is found in a ICA component, the latter is processed with an adaptive Wiener filter with the objective of estimating the artifact component in the ICA segment. The estimated contamination is subtracted from the ICA component in question and EEG signals are reconstructed. This method allows removing artifacts from EEG with small distortion and loss of physiological information [55]. Finally, reconstructed EEG of each trial is inspected visually to verify its quality and noisy trials are excluded from the analysis.

Once preprocessing is performed, the EEG of every trial is processed with a spatial filter that subtracts from C3, Cz and C4 the mean of their four neighbouring electrodes. The electrodes considered for C3 are FC5, FC1, CP5, and CP1, while for Cz are FC1, FC2, CP1, and CP2, and the ones for C4 are FC2, FC6, CP2, and CP6. The output of these three channels is divided in a MI epoch and its corresponding rest EEG epoch and also labeled with the kind of MI that was performed at the trial, so EEG epochs of MI and its rest period are separated for right hand and feet MI. In the case of the stimulation modality in which tDCS is applied during EEG acquisition, the first 4 s of the rest state are omitted. After obtaining the signal epochs, spectra after the second 2 for every epoch of MI and rest is calculated. Then, the same procedure is carried out for each kind of MI (hand and feet), in which the epochs corresponding to the EEG information at Cz, C3 and C4 is used to calculate the accuracy of classification between rest and MI events, as well as the ERS changes associated to the performance of MI at each session. These three channels were selected for the analysis because they are located on the neighborhood of the motor areas that may present a higher impact after tDCS, considering that C3 is located over the right hand motor cortex area and Cz is over the feet motor cortex region [56]. In addition, there is evidence of EEG connectivity between C3 and C4 at some frequencies and it has also been reported an increasing ERS surrounding a region of EEG desynchronization during motor imagery for some subjects [57]. The relevance of these channels is supported by the observation of ERS changes at their location during right or left hand and foot motor imagery [58], as well as their use in motor imagery analysis [59, 60]. The process performed for estimating the accuracy and ERS is described next.

- **Accuracy:**
  Fisher criterion (*F*) [61] of the spectra of MI and rest states in C3, C4 and Cz at 9-30 Hz is calculated as follows:
  1 $$\begin{array}{@{}rcl@{}} F=\frac{\left(\mu_{1f}-\mu_{2f}\right)^{2}}{\delta_{1f}^{2}+\delta_{2f}^{2}}, \end{array} $$
  where *μ*_1*f*_ and *μ*_2*f*_ denote the mean power at frequency *f* for conditions 1 (MI) and 2 (rest), respectively, while *δ*_1*f*_ and *δ*_2*f*_ correspond to the standard deviation of power at frequency *f* for conditions 1 and 2, respectively. Then, the maximum value of *F* for the evaluated frequency range is determined for C3, Cz and C4, so a characteristic frequency is obtained for each of the three channels in every session. This characteristic feature represents the frequency in which the MI and rest states show a greater difference in their means and lower variance, which means both conditions are more discernible. Then, 100 iterations are conducted in which 30 random epochs are selected from the total epochs of both MI and rest and then they are labeled as either MI or rest with a linear discriminant analysis (LDA) classifier. This classifier is trained with the spectral power of C3, Cz and C4 of the rest of the epochs of the session at the characteristic frequency of these channels. Once the classification is performed, the percentage of correct classification of the 30 epochs is calculated per iteration. After completing the iterations, a distribution of the accuracy with a size of 100 samples is obtained for the session.
  It should be mentioned that the methodology that was described previously is focused on obtaining individual features at each session. Subject-specific features were calculated based on the approach of BCI technology personalization due to high inter-subject variability [62]. In addition, features were obtained for each session in order to take into account intra-subject variability of the sensorimotor rhythm modulation, which is the main reason for which MI-based BCIs include multiple training sessions [63].
- **ERS:**
  ERS on C3, Cz and C4 is obtained for each trial as the difference of the natural logarithms of the mean spectral power during MI and the mean spectral power during rest of *μ* or *β* band. The logarithm is used in this case to reduce skewness of ERS distribution. Considering that *S*_1_(*f*) and *S*_2_(*f*) represent the spectral power of conditions 1 (MI) and 2 (rest), respectively, at frequency *f*, which is within *μ* or *β* band, ERS is calculated as:

2 $$\begin{array}{@{}rcl@{}} ERS_{\mu}=\ln{\sum\limits^{f=12}_{9}\frac{S_{1}(f)}{4}}-\ln{\sum\limits^{f=12}_{9} \frac{S_{2}(f)}{4}} \end{array} $$

3 $$\begin{array}{@{}rcl@{}} ERS_{\beta}=\ln{\sum\limits^{f=30}_{13}\frac{S_{1}(f)}{18}}-\ln{\sum\limits^{f=30}_{13} \frac{S_{2}(f)}{18}}, \end{array} $$

After the values of accuracy and ERS are obtained for all sessions, statistical comparisons can be made between current densities. In the case of ERS, possible outliers are excluded from data of each session for further statistical analysis. This is done by discarding data that is out of the 95% confidence interval, according to the method described in [56] to determine significant spectral changes. Also, for band power analysis, mean ERS of sham sessions are subtracted from all sessions of the same tDCS modality in order to set sham session as a reference of ERS with zero value. Then, Barlett tests (*p*>0.05) were performed to confirm that about 70% of the sessions from all users can be assumed to have homogeneous variance if the variances of the sessions of the same user and stimulation scheme are compared. In the case of accuracy, analysis of variance (ANOVA) tests (*p*<0.001) are performed in order to identify significant statistical differences in accuracy between sessions of the same tDCS modality for each subject. When significant differences are detected, multiple comparisons are made using Tukey-Kramer’s method (*p*<0.001). In the case of ERS, t-tests (*p*<0.05) are performed only for the stimulation schemes when significant improvements are found for accuracy to compare just sham session with results from other current densities. Significance threshold is set as 0.001 for accuracy to assure that the probability of detecting improvements on classification is lower, as we consider the use of tDCS to have a high cost. ERS analysis is used for providing a greater scope about the differences in EEG that could enhance good classification.

## Results

### tDCS simulation

**Fig. 5:**
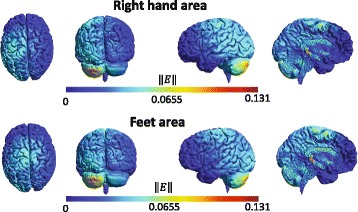
Simulation of tDCS. Simulation of the norm of the electric field (∥*E*∥, in V/m) induced by stimulation over *right-hand* (*top*) and *feet* (*bottom*) motor area. From *left* to *right*, *superior*, *posterior* and *right side* lateral views of the brain and an image of the *left* hemisphere are presented

In Fig. 5, from right to left, we can see a sagittal view of the left hemisphere, sliced slightly right from the longitudinal fissure, a lateral view of the left hemisphere, and a posterior and dorsal view of the whole brain. There, the effects of a current intensity of 180 *μ*A, which is approximately the maximum current applied during the stimulation, can be appreciated for an electrode size of *π* cm^2^. As it can be seen from the dorsal view, despite the electrode diameter, the current propagates both towards SMA and somatosensory cortex with an approximately homogeneous intensity. Proceeding caudally on the cortex surface, the current almost fades in the parietal region, strengthening in the occipital area. In addition, it can be found a higher electric field ∥*E*∥ affecting mainly the left cerebellum. Note that both montages present stimulation near the sensorimotor area lying inside the longitudinal fissure, but stronger for the feet motor area stimulation. This is a desired behaviour for feet motor area stimulation, since feet motor cortex is placed deep in the fissure, which makes it difficult to be targeted [64]. For both versions of the montage it should be considered that other nearby motor areas are affected, but not necessarily with significant effects. From the sagittal view it can be seen a region where there is higher current intensity. The location of this area is close to the red nucleus and the thalamus, which are part of a neural pathway that connects cerebellum toward cortical motor areas [37]. It should be noticed that some studies reveal that there is a neuroanatomical closed loop pathway that links the motor cortex with the cerebellum through the thalamus [65], but there are still few pioneer studies that investigate the possible tDCS effects on subcortical structures. Nevertheless, [66] reported that anodal tDCS applied over the primary motor cortex is capable of modifying the cortico-striatal-thalamo-cortical motor circuit. Therefore, electric field hot spots at different parts of the pathway between cerebellum an cortical areas may suggest an effect at various components of such path and that the current flow may be following a similar orientation. Yet, the cumulative effect of affecting different anatomical areas is uncertain. Moreover, affecting parts of the path that carries the projections from different cortical areas may have nonspecific effects. Regarding to the similarity of orientation between the current flow and the cerebello cortical path, it seems that the montage version that stimulates the right-hand motor area may target better the orientation of the neural path in [36, 37], considering that in such study the cortical projections are grouped and directed to inner structures following visually a diagonal direction to the midline in relation to the motor cortex of each brain hemisphere.

### Accuracy

**Fig. 6:**
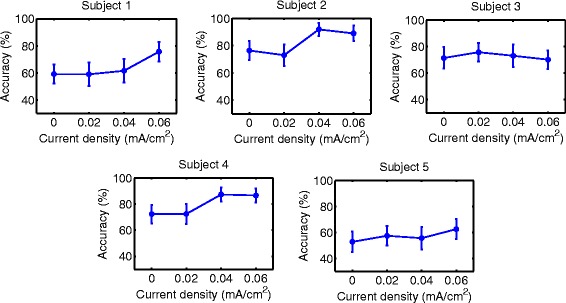
Accuracy of *right hand* MI with tDCS applied over *hand* area before EEG recording. Mean accuracy is presented (*circular markers*) for all subjects and current densities. *Error bars* represent standard deviation of accuracy

**Fig. 7:**
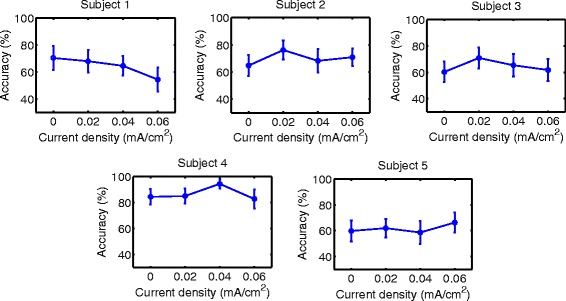
Accuracy of *feet* MI with tDCS applied over *hand* area before EEG recording. Mean accuracy is presented (*circular markers*) for all subjects and current densities. *Error bars* represent standard deviation of accuracy

**Fig. 8:**
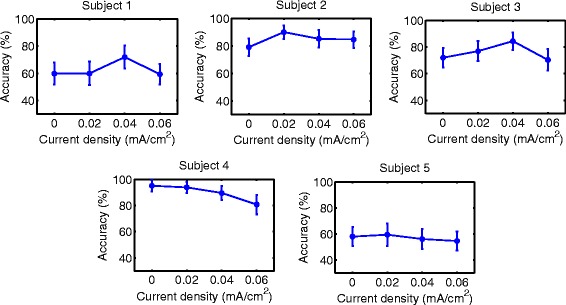
Accuracy of *right hand* MI with tDCS applied over *feet* area before EEG recording. Mean accuracy is presented (*circular markers*) for all subjects and current densities. *Error bars* represent standard deviation of accuracy

**Fig. 9:**
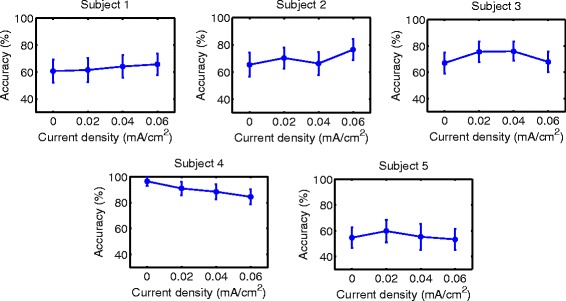
Accuracy of *feet* MI with tDCS applied over *feet* area before EEG recording. Mean accuracy is presented (*circular markers*) for all subjects and current densities. *Error bars* represent standard deviation of accuracy

**Fig. 10:**
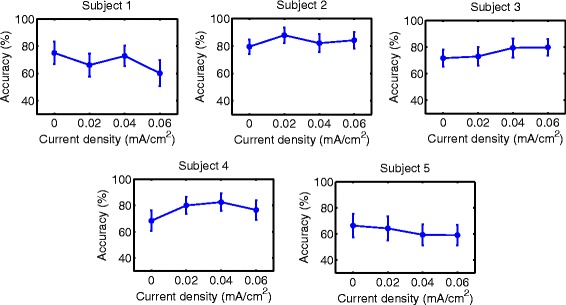
Accuracy of *right hand* MI with tDCS applied during EEG recording. Mean accuracy is presented (*circular markers*) for all subjects and current densities. *Error bars* represent standard deviation of accuracy

**Fig. 11:**
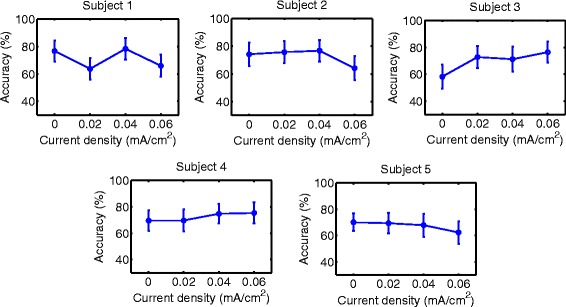
Accuracy of *feet* MI with tDCS applied during EEG recording. Mean accuracy is presented (*circular markers*) for all subjects and current densities. *Error bars* represent standard deviation of accuracy

Accuracy results are shown in Figs. 6, 7, 8, 9, 10 and 11 for each type of MI, subject and stimulation scheme, while statistical analysis are shown in Tables 1, 2 and 3. There, first and second columns specify the type of MI, i.e., right hand (H) or feet (F), and the number of subject that is being examined. The third and fourth columns show the results of statistical tests, which include the *p*-value from ANOVA and comparisons between the different current densities. For example, in Table 1 for Subject 2 and right hand MI, providing sham (D0) or 0.02 (D1) mA/cm^2^ stimulation resulted in a lower accuracy compared to the supply of 0.04 (D2) or 0.06 (D3) mA/cm^2^. This relation is written as D0, D1 < D2, D3. Description of accuracy results is developed for each stimulation modality.

**Table 1:** Statistical tests for accuracy using tDCS over right hand area before EEG recording

MI	S	ANOVA	Multiple comparisons
		(*p*-value)	(*p* <0.001)
H	1	7.26×10^−49^	D0, D1, D2 <D3
	2	1.83×10^−80^	D0, D1 <D2, D3
	3	3.00×10^−6^	D0, D3 <D1
	4	2.77×10^−71^	D0, D1 <D2, D3
	5	1.55×10^−15^	D0 <D1 <D3;
			D2 <D3
F	1	6.26×10^−36^	D3 <D2 <D0;
			D3 <D1
	2	3.09×10^−23^	D0 <D3 <D1;
			D2 <D1
	3	3.27×10^−19^	D0 <D2 <D1;
			D3 <D1
	4	7.24×10^−42^	D0, D1, D3 <D2
	5	7.13×10^−11^	D0, D1, D2 <D3

**Table 2:** Statistical tests for accuracy using tDCS over feet area before EEG recording

MI	S	ANOVA	Multiple comparisons
		(*p*-value)	(*p* <0.001)
H	1	4.53×10^−30^	D0, D1, D3 <D2
	2	5.17×10^−30^	D0 <D2, D3 <D1
	3	2.69×10^−38^	D0, D3 <D1 <D2
	4	2.81×10^−62^	D3 <D2 <D0, D1
	5	6.77×10^−5^	D3 <D1
F	1	5.99×10^−5^	D0 <D3
	2	8.73×10^−22^	D0 <D1 <D3;
			D2 <D3
	3	1.10×10^−21^	D0, D3 <D1, D2
	4	1.03×10^−45^	D3 <D1, D2 <D0
	5	2.08×10^−6^	D0, D3 <D1

**Table 3:** Statistical tests for accuracy using tDCS over right hand or feet area during EEG recording

MI	S	ANOVA	Multiple comparisons
		(*p*-value)	(*p* <0.001)
H	1	1.26×10^−33^	D3 <D1 <D0, D2
	2	1.72×10^−20^	D0 <D3 <D1;
			D2 <D1
	3	1.29×10^−22^	D0, D1 <D2, D3
	4	1.08×10^−38^	D0 <D1, D2, D3;
			D3 <D2
	5	5.47×10^−11^	D2, D3 <D0, D1
F	1	1.81×10^−44^	D1, D3 <D0, D2
	2	3.32×10^−27^	D3 <D0, D1, D2
	3	8.25×10^−43^	D0 <D1, D2, D3;
			D2 <D3
	4	8.40×10^−10^	D0, D1 <D2, D3
	5	1.32×10^−11^	D3 <D0, D1, D2

#### Supply of tDCS over the brain region related to right-hand movement before EEG recording

Results for this tDCS scheme are shown in Table 1, as well as Figs. 6 and 7 for right hand and feet MI, respectively. There it can be observed that for 4 out of 5 subjects, D3 could improve the classification performance of right-hand motor imagery compared to D0. Moreover, the user that had a different behavior (S3) did not show a significant different accuracy for D3 compared to D0, according to the statistical analysis in Table 1. This can be considered a beneficial trend on accuracy, since the outcomes of the stimulation were either the presence of no significant changes or accuracy improvements for most volunteers. In particular, the mean accuracy improvements for Subjects 1, 2, 4, and 5 were 16.50, 12.53, 14.26, and 9.77%, respectively. This represents an improvement of about 10% for the participants. On the other hand, Subject 3 showed a non significant decrease of 1.26%, which could be attributed to random variability of the accuracy, according to the statistical analysis.

In contrast, for the case of feet motor imagery, it can be seen that no current density improved accuracy for most subjects.

#### Supply of tDCS over the brain region related to feet movement before EEG recording

Figures 8 and 9, in addition to Table 2, present the results of this stimulation mode. For both kinds of MI, there was not a single current density value within the evaluated range that improved the classification performance for most subjects without affecting negatively the accuracy of some subject.

#### Supply of tDCS over the brain region related to right-hand or feet movement during EEG recording

Figures 10 and 11, along with Table 3, show the accuracy and statistical results for the case of applying tDCS of short duration during EEG recording. For both right-hand and feet MI, there are no distinguishable favorable trends on accuracy with the various current densities.

In summary, the only tDCS modality that had a possible beneficial trend on accuracy within the evaluated current values was the scheme that supplied stimulation for 10 minutes over the right hand motor area before EEG recording. In particular, the current density that provided the best results was D3. This current density improved accuracy for about 10% on 4 out of 5 users, while the remaining subject showed no significant effects due to the stimulation.

### ERS analysis

**Fig. 12:**
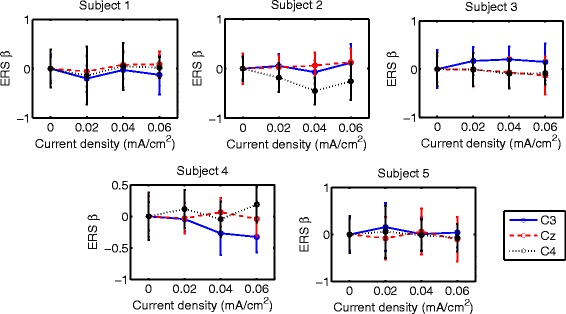
*β*-ERS during *right hand* MI with tDCS applied over *hand* area before EEG recording. Mean ERS is presented (*circular markers*) for all subjects and current densities in C3, Cz and C4. *Error bars* represent standard deviation of ERS

Table 4 includes the t-tests that compare ERS results of 0 mA/cm^2^ and other current densities for the only case where it seemed to exist an improvement trend on classification, which was for right-hand MI when tDCS was applied over the right-hand motor area. The first and second columns indicate the number of subject and the channel that are examined, respectively. Result of ERS comparisons between 0 mA/cm^2^ tDCS and other current densities are shown on the last two columns for *μ* and *β* bands. For example, for the case of subject 2 when tDCS was applied over right-hand brain area, it is found that ERS on C4 of *μ* and *β* bands is lower for 0.02 (D1), 0.04 (D2) and 0.06 (D3) mA/cm^2^ than for 0 mA/cm^2^ (D0). This is expressed as D1, D2, D3 <D0 and it means that D1, D2 and D3 showed a higher attenuation or desynchronization than D0. For simplicity, description of ERS changes will be limited to those current values for which accuracy seemed to improve. Note that ERS statistical results were obtained from data with high variance. As a representative result, Fig. 12 shows *ERS*_*β*_ for right-hand MI when right-hand motor region was stimulated. There, mean ERS and standard deviation for all users on C3, Cz and C4 for the various current densities are presented. As can be seen, variance is high. Considering that significant statistical differences were found, it must be considered that subtle ERS changes can be statistically significant. Nevertheless, t-test results are useful for ERS trend identification.

**Table 4:** Statistical tests for ERS using tDCS over right hand area before EEG recording

S	Channel	*μ* band	*β* band
1	C3	D1, D2 <D0	D1 <D0
	Cz	-	D3 >D0
	C4	D1, D2 <D0	-
2	C3	D1, D3 >D0	-
	Cz	-	D3 >D0
	C4	D1, D2, D3 <D0	D1, D2, D3 <D0
3	C3	D1 >D0	D1, D2, D3 >D0
	Cz	D3 <D0	D3 <D0
	C4	-	D3 <D0
4	C3	D1 >D0	D2, D3 <D0
	Cz	D1 >D0	-
	C4	D1, D2, D3 >D0	D1, D3 >D0
5	C3	-	-
	Cz	-	-
	C4	-	-

In the case of the tDCS modality in which the right-hand motor area was stimulated, the current density that could improve accuracy of right-hand MI was D3. In order to facilitate reading, significant ERS changes are described for each subject:

- *S1*: This subject presented higher *ERS*_*β*_ over Cz when D3 was applied. This synchronization change may be considered beneficial for right-hand MI, since it is associated to the decrease of activity on the feet motor area, which can represent the inhibition on surrounding areas of the right-hand motor cortex. This ERS change is consistent with the higher accuracy that was achieved with that current value.
- *S2*: This user exhibited changes that are not considered to benefit right-hand MI, such as higher *ERS*_*μ*_ on C3 for D3 and lower ERS on C4 in *μ* and *β* bands for the same current.On the other hand, higher *ERS*_*β*_ on Cz was found when D3 was supplied, which represents the same advantageous changes observed for S1. These last effects are in accordance with the higher accuracy that was reached when D3 is applied.
- *S3*: This participant showed no significant changes that could improve MI. Higher *ERS*_*β*_ on C3 was observed with D3. Also, lower *ERS*_*μ*_ and *ERS*_*β*_ on Cz was found after D3 stimulation, as well as lower *ERS*_*β*_ on C4. Considering that this user showed no significant changes in accuracy, it seems that for this subject there is no clear relation between accuracy and ERS with the protocol of this study, even if this subject actually showed a slight accuracy decrement with this stimulation intensity that may seem consistent with the ERS results.
- *S4*: Subject 4 had several ERS changes that could reinforce MI. In particular, *ERS*_*β*_ on C3 was lower when D3 was applied. In addition, higher *ERS*_*μ*_ and *ERS*_*β*_ were observed on C4 with D3. This volunteer had an improvement on EEG classification after stimulating with D3, which is congruent with all ERS changes.
- *S5*: No synchronization changes were found for D3, even though this user presented a showed a classification improvement of right-hand MI with this current value. However, it should be noted that this user was the one with the smallest accuracy improvement.

In general, there is no direct relation between ERS changes and accuracy for the used protocol. The only case with a favorable change in classification was for right-hand MI when the right-hand motor area is stimulated with D3, which is the highest evaluated current that was evaluated in this study. This current value seemed to affect different parts of the motor area variably among subjects. Three of the subjects (1, 2 and 4), who presented accuracy improvements above 10% presented at least one ERS change that could be considered beneficial for right-hand MI enhancement, while the user that presented the smallest classification improvement (subject 5) did not presented significant ERS changes. In contrast, the user that showed a non significant decrement on accuracy exhibited only ERS changes that do not appear to enhance right-hand MI. Interestingly, two of the users showed a significant higher motor activity over C4 at either *μ* of *β* bands, while only one had it for C3 at the *β* band. This is associated to higher activity over the right motor cortex.

## Discussion

As accuracy results show, the only tDCS scheme that could improve accuracy was the one that supplied tDCS for 10 minutes over the right-hand motor area before recording EEG, just for right-hand motor imagery classification. In particular, four out of five subjects improved their accuracy of classification about 10%, which could represent a valuable increment considering that the accuracy threshold that is usually considered as an indicator of enough BCI control is of 70% for a two choice system [67, 68] with a chance level of 50%. It should be mentioned that these improvements where found for the maximum evaluated current, i.e. 188 *μ*A (for an approximate current density of 0.06 mA/cm^2^). Taking into account that the foot motor cortex is more difficult to target because of its position at the longitudinal fissure, it is probable that stimulation of this area with the montage would require a higher current intensity, which may explain the lack of effect for the montage version that was aimed to stimulate the feet motor area.

When the experimental protocol was first designed, effects for at least one of the evaluated current values were expected, since it was previously reported in [52] that the reduction of the electrodes up to 3.5 cm^2^ showed no significant difference on the tDCS effects compared to when bigger electrodes are used, as long as the current density was kept constant. In that study, the smallest size of the electrode is similar to the one used in this study (*π* cm^2^). Also, the current density was kept at approximately 0.03 mA/cm^2^ and estimated as the current intensity and electrode size ratio [52]. This ratio was also used in this study to calculate the current strength that was necessary to provide the desired current density. However, the latter was apparently overestimated, according to [51], which indicates that higher currents have to be provided with smaller electrodes in order to achieve comparable current densities respect to when bigger electrodes are used. The inaccuracy in the approximation of the current values suggests that higher intensities of stimulation were needed in order to observe effects related to the supply of tDCS with both montage versions.

It must be considered that the stimulation scheme that provided tDCS for 4 s over the right-hand motor area may have not got similar results in the case of right-hand MI for different reasons, besides the tDCS of short duration not being adequate. One of the possible reasons is that even though the tDCS pulse lasts only 4 s, the real timing of the stimulation cue lasts 16 s because of the stimulation device. Then, the subjects had to wait about 20 s between the performance of each MI, which may affect the attention level that the users had during the task. Moreover, the time lapse that was implemented for the attenuation of tDCS effects was based on the duration of the effects with another cephalic montage with a current density that was similar to the lowest current density that was applied (0.02 mA/cm^2^) in this study. Hence, whether the effects were significant or they faded at the end of the recovery period is unknown. In addition, such period includes the first seconds of the rest state, so the highest synchronization difference after MI performance was probably discarded from the analysis. In consequence, an adequate protocol for the stimulation of short duration may be proposed after measuring the approximate time that the tDCS effect lasts with the montage. Also, signal processing would help in decontaminating EEG samples that have noise because of the activation or deactivation of the stimulation device, so the time the user has to wait between each MI could be reduced, making the experimental session shorter and less demanding for the subject. However, the current state of this short-time tDCS protocol regarding to the time required to complete each session makes the tDCS modality useless for further evaluation.

The simulation of the electric field indicated that different parts of the motor path between the cerebellum and cortical areas may be affected for both versions of the proposed montage approach. Also, it seems that the electric field at the cortical areas is disperse, so different motor areas may be impacted by the stimulation, but not necessarily with significant effects. Moreover, the possible outcomes of affecting the cerebellum were not completely clear since studies about cerebellar tDCS report variable results [26]. ERS analysis showed that some users had EEG activity changes over the right motor cortex that are related to increment of motor activity, which may be due to CBI suppression caused by inhibition at the left cerebellum. This ERS variability over the right motor cortex area possibly indicates the need of improving the positioning of the electrode that affects the cerebellum, which could involve consideration of anatomic details of the subject, for increasing reproducibility of the results [69]. Nevertheless, evaluation of the proposed montage provided some insight of the current intensity that may cause observable effects on MI detection before any further evaluation of the montage with a large sample of users is performed.

Whether the cerebellar approach can indeed prevent compensatory effects, as firstly hypothesized, would possibly require the evaluation of the montage during motor-sequence learning. For the tasks of one session without feedback that were evaluated in this study, there is no evidence that this might occur. On the other hand, ERS changes could not be related directly to accuracy changes because subject-specific features for classification where chosen in each session in order to account for intra-subject variability. This is a limitation of the study because the ERS analysis cannot describe satisfactorily the EEG changes that may lead to accuracy improvement. In this case, evaluation of the montage during BCI training would be useful for the reason that brain entrainment is suspected to minimize feature variability over the experimental sessions [70], which could facilitate ERS analysis. It should be noted that not all subjects have the skills for performing detectable desynchronization patterns in their EEG during MI without training [71], so brain entrainment would offer more stable EEG classification features and the possibility of evaluating the montage in a learning context that represents better the conditions in which the tDCS montage was expected to exhibit its hypothesized behavior.

At this moment, it must be considered that this study allows to describe some qualitative results of the proposed montage that, after a more extensive characterization of its possible best combination of parameters, could be implemented to enhance and study MI detection, which is of interest in the field of MI-based BCIs in neurorehabilitation. Future work would include evaluation of the montage with higher currents, focusing on lower limb MI, which is the main interest of the research project. Note that the sample size of the study was small, so further experiments should be developed with more subjects when a current range is suspected to benefit MI detection.

Even though cerebellar stimulation effects were attractive to be studied, along with other areas such as SMA and motor cortices, the real weaknesses or opportunities related to this montage cannot be known until comparisons with other montages are performed. Hence, as future work, we also plan to make a comparison with a montage that stimulates just the motor cortex.

## Conclusions

It was proposed an anodal tDCS montage that affects the motor cortical areas and cerebellum, which would influence different parts of the cerebello-cortical pathway, with the aim of improving the performance of motor tasks. Based on the analysis of accuracy, it seems that the proposed montage has potential to increase MI detection in healthy users, which is of interest for its possible future use in MI-based BCIs in neurorehabilitation, where users with heterogeneous brain conditions may be benefited by the stimulation of several motor brain structures. Results showed that with the maximum current that was evaluated in this study (188 *μ*A) only right-hand MI detection seemed to be improved when tDCS is applied 10 minutes before recording the EEG signals that are used for classification. In order to explore the possibility of improving feet MI with the stimulation of the feet motor areas, it is probable that higher current values are required.

Band power analysis indicates that ERS changes cannot be related directly to classification improvement, which is a limitation of the study, so strategies for improving the analysis of EEG in relation to accuracy changes need to be implemented. For example, including in the experimental protocol brain entrainment sessions that reduce the intra-subject variability of classification features and, thus, facilitate analysis of EEG changes. This approach could also describe better the neuroplasticity effects of the montage. However, higher currents need to be tested first to define the best current range to perform montage evaluation, in particular for lower limb motor imagery.

In addition, the actual potential of the tested montage could be confirmed just when future comparisons with other montages are performed. Hence, long-term future work would also include a comparison of the presented montage with one that stimulates just the motor cortex, so that the cerebellar stimulation effects are cut off.

## Abbreviations

ANOVA: Analysis of variance
CBI: Cerebellar brain inhibition
EEG: Electroencephalography
ERS: Event-related synchronization
ICA: Independent component analysis
LDA: Linear discriminant analysis
M1: Primary motor area
MEP: Motor evoked potential
MI: Motor imagery
PM: Premotor area
SMA: Supplementary motor area
tDCS: Transcranial direct current stimulation
